# Airway Morphometric Changes Following Prefabricated Myofunctional Appliance in Class II Division 1 Patients: A Clinical Evaluation

**DOI:** 10.3390/life16060911

**Published:** 2026-05-28

**Authors:** Liang-Ru Chen, Chia-Li Lai, I-Chieh Chen, Jun-Peng Chen, Ming-Ju Lee

**Affiliations:** 1 School of Dentistry, Yang Ming Chiao Tung University, Taipei 11221, Taiwan; 2 School of Dentistry, College of Oral Medicine, Chung Shan Medical University, Taichung 40201, Taiwan; 3 Division of Pediatric Dentistry and Orthodontics, Department of Stomatology, Taichung Veterans General Hospital, Taichung 40705, Taiwan; claytonbuzz963@gmail.com; 4 Department of Medical Research, Taichung Veterans General Hospital, Taichung 40705, Taiwan; icchen@vghtc.gov.tw (I.-C.C.); pippan7676@vghtc.gov.tw (J.-P.C.); 5 Master’s Program in Precision Health, National Chung Hsing University, Taichung 40227, Taiwan

**Keywords:** Class II division 1 malocclusion, prefabricated myofunctional appliance, airway morphology, pediatric orthodontics, sleep-disordered breathing

## Abstract

Prefabricated myofunctional appliances (PMAs) are designed to improve airway function by advancing the mandible, enhancing tongue posture, and reducing airway resistance, thereby facilitating nasal breathing in children with sleep-disordered breathing (SDB). This retrospective study evaluated the effects of PMAs on airway dimensions in children with skeletal Class II division 1 malocclusion. Patients were selected from a departmental database (2017–2019). The treatment group included children with Class II division 1 malocclusion, an incisor overjet of ≥6 mm, cervical vertebral maturation (CVM) stage III or earlier, and documented myofunctional dysfunction (e.g., adenoid hypertrophy, allergic rhinitis, or mouth breathing), with complete pretreatment and one-year follow-up lateral cephalometric radiographs. Patients with prior orthodontic intervention or poor compliance were excluded. A matched observation group consisted of untreated patients undergoing growth monitoring. Airway dimensions of the nasopharynx, oropharynx, and hypopharynx were measured using cephalometric radiographs, along with McNamara Airway Analysis. The total nasal symptom score (TNSS) was used as a self-report measure. A total of 34 patients (mean age 9.4 years) were included in the PMA group and 29 patients (mean age 9.6 years) in the observation group. Compared with controls, the PMA group demonstrated significant increases in nasopharyngeal (*p* = 0.044) and oropharyngeal (*p* = 0.039) airway areas, while changes in the hypopharyngeal area were not significant (*p* = 0.121). McNamara Airway Analysis also showed a significant improvement in upper pharyngeal airway dimensions (*p* = 0.018). TNSS revealed significant changes following PMA therapy (*p* < 0.001). These findings indicate that PMA therapy is associated with enlargement of the nasopharyngeal and oropharyngeal airway in children with skeletal Class II division 1 malocclusion, suggesting functional airway adaptation beyond simple mandibular advancement.

## 1. Introduction

Skeletal class II malocclusion is a common dentofacial abnormality characterized by mandibular retrognathism, maxillary prognathism, or a combination of both [1,2]. Within this classification, Class II division 1 is distinguished by proclination of the maxillary incisors, resulting in compromised facial aesthetics and functional disturbances. Besides clinical concerns, patients exhibiting this type of malocclusion present with anatomical characteristics that predispose them to sleep-disordered breathing (SDB) [3]. These include mandibular retrusion, posterior tongue position, a high and narrow palatal vault, a hyperdivergent facial profile, and increased overjet [4]. Consequently, individuals with Class II division 1 malocclusion may be at an elevated risk for SDB [5]. Accordingly, the established relationship between craniofacial morphology and airway patency emphasizes the clinical significance of early recognition and intervention to reduce the likelihood of long-term respiratory complications.

Although cone beam computed tomography (CBCT) provides more accurate three-dimensional airway measurements, lateral cephalometric radiographs are still frequently used in image studies due to radiation exposure concerns in pediatric patients and their routine use in orthodontic assessment. Functional appliances (FAs) and prefabricated myofunctional appliances (PMAs) are usually used in children with Class II division 1 malocclusion. These appliances are designed to advance the mandible and facilitate neuromuscular adaptation, thereby creating a more favorable functional environment for craniofacial growth. Commonly used FAs include Bionator, Activator, Twin Block appliance, and Frankel appliances. Commonly used PMAs include EF Orthoplus, Myobrace, and LM-Activator. PMAs are different from FAs in the following aspects: (1) ready-made; available in various sizes; (2) mainly worn at night; (3) more comfortable to use due to silicone or polyurethane; (4) can be used simultaneously with myofunctional exercises such as breathing, swallowing, tongue, and lip strength training. Systematic reviews have shown that both appliances induce dentoalveolar changes, such as overjet correction and skeletal improvement [6,7,8,9]. In addition to dentoalveolar and skeletal effects, FAs and PMAs are thought to improve airway dimensions and breathing conditions due to mandibular advancement and a forward-positioned tongue.

Vinoth et al. evaluated the effect of the Twin Block appliance on airway dimensions in growing class II patients using cephalometric radiographs [10]. They observed a significant increase in upper and lower pharyngeal width. Han et al. evaluated the effect of Bionator on pharyngeal airway changes [11]. They concluded that Bionator therapy not only increased pharyngeal airway dimensions but also maintained this effect until the completion of growth. Kannon et al. reviewed the effect of functional appliances on the airway dimensions in patients with skeletal class II malocclusion [12]. They concluded that the FAs increased the airway dimensions at different sections of the airway. Another systematic review and meta-analysis concluded that FAs enlarged airway dimensions mainly in the oropharyngeal region, in growing subjects with skeletal class II malocclusion [13].

Median and Dina Elfouly’s study compared the airway changes between Twin Block and Myobrace [14]. However, without a normal population or observation group as a control, the true effect of both appliances could not be verified.

The study aims to examine the effects of PMAs on airway changes and compare them with those in untreated class II division I children, to elucidate the clinical implications of airway management and potential integration into interdisciplinary treatment strategies.

## 2. Materials and Methods

### 2.1. Patient Selection

This study was designed as a retrospective controlled study. The patient population was drawn from patients who sought care in the Department of Pediatric Dentistry and Orthodontics at the Taichung Veterans General Hospital.

#### 2.1.1. Selection Criteria for Children with Angle Class II Division 1 Malocclusion

Inclusion Criteria:

- Diagnosis of Angle Class II division 1 malocclusion.
- Overjet ≥ 6 mm.
- Cervical vertebral maturation (CVM) stage III or earlier.
- A treatment period of more than one year with the EF appliance.
- Availability of cephalometric radiographic records before treatment and one year after treatment.
- Completed total nasal symptom score (TNSS) at the beginning and the end of the observation.

Exclusion Criteria:

- Previous orthodontic interventions.
- Poor appliance compliance, defined as daily wear of <7 h or <5 days per week.
- Incomplete cephalometric radiographs at baseline or one-year follow-up.
- Incomplete TNSS score data.
- Presence of congenital craniofacial abnormalities or systemic diseases.

#### 2.1.2. Selection Criteria of Observation-Only Group: The Data Were Retrieved from Departmental Records Data (2017–2019) with the Following Criteria

- Angle Class II division 1 malocclusion.
- Overjet ≥ 6 mm.
- No history of interceptive orthodontic treatment.
- Availability of initial and one-year follow-up cephalometric radiographs.
- CVM stage III or earlier.
- No congenital craniofacial anomalies or systemic diseases

### 2.2. Selection and Use of Myofunctional Appliances and Exercise Protocol

**Figure 1:**
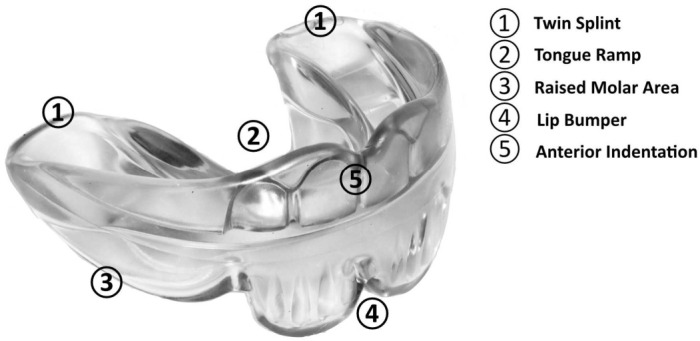
Prefabricated myofunctional appliance Functional education (EF) (OrthoPlus, Igny, France).

The myofunctional appliance used in this study was the Education Fonctionnelle (EF) (OrthoPlus, Igny, France) (Figure 1). The appliance size was selected from the patient’s dental cast in accordance with the manufacturer’s guidelines. Children with Angle Class II division 1 malocclusion were treated using the EF Class II appliances. Children with Angle Class II division 1 malocclusion were treated using the EF Class II appliances.

Patients were instructed to wear the appliance for at least 8 h per day, including approximately 2 h of daytime use combined with breathing-related myofunctional exercises and overnight wear during sleep. For patients presenting with tongue dysfunctions such as low tongue posture or tongue thrust during swallowing—additional targeted exercises were prescribed, including water-swallowing training and tongue-muscle-strengthening protocols.

Before initiating the treatment, the patients were required to undergo the following diagnostic procedures:

1. Panoramic radiography and cephalometric radiographs.
2. Extraoral and intraoral photographs.
3. Study models (dental casts).

The cephalometric radiographs were taken using ORTHOPHOS XG 3D CEPH (Bensheim, Germany).

### 2.3. Standardization of Cephalometric Acquisition and Head Positioning

Airway measurements in this study were obtained from lateral cephalometric radiographs acquired in natural head position. All radiographs were repeatedly printed and rescaled until a true 1:1 ratio was achieved to ensure measurement accuracy.

### 2.4. Airway Measurement

Patients in both the treatment and observation groups attended follow-up appointments at 3-month intervals, in accordance with standard departmental protocols. In the treatment group, each visit included the assessment of appliance fit, monitoring for mucosal or dental discomfort, and review of the wear time log and compliance with muscle training exercises.

**Figure 2:**
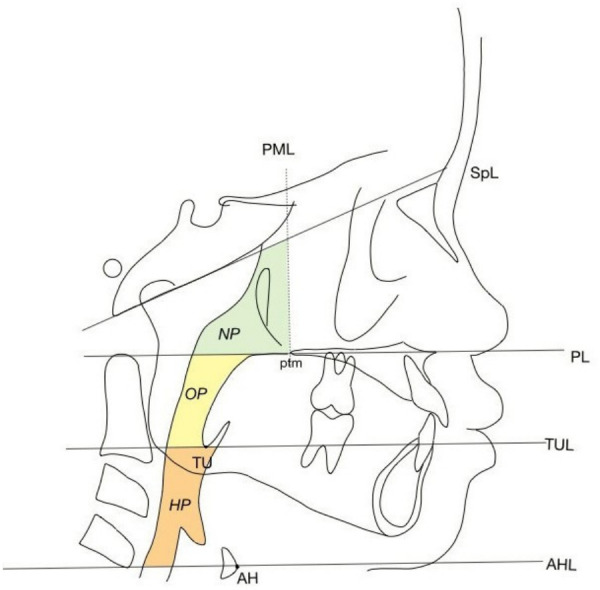
Definition of the nasopharynx (NP), oropharynx (OP), and hypopharynx (HP) based on cephalometric landmarks. The regions are delineated using reference lines, including: the Sphenoid line (SpL), tangent to the lower border of the sphenoid registered on basion; the Pterygomaxillary line (PML), perpendicular to the palatal line and registered on the pterygomaxillary point (ptm); the Palatal line (PL); the Tip of Uvula line (TUL), registered on the tip of the uvula (TU) and parallel to the palatal line; and the Anterior Hyoid line (AHL), registered on the most anterior point of the hyoid bone (AH) and parallel to the palatal line. The dotted line between PML and ptm represents the reference line used for cephalometric measurement. The marked areas from top to bottom, shown in green, yellow, and orange, correspond to the nasopharynx (NP), oropharynx (OP), and hypopharynx (HP), respectively.

In the observation group, oral examination, oral hygiene instruction, and occlusal changes were performed at each visit. Orthodontic data collection was conducted annually. Definition of airway measurement from cephalometric measurements (Figure 2):

(A) Nasopharyngeal airway area (cm^2^): the area was formed by a trapezoid-like area with inferior border formed by Ptm (pterygomaxillare) extending to most posterior border of pharyngeal wall along the palatal line (ANS-PNS) extension; posterior border formed by the posterior wall of pharynx; anterior wall formed by the PML (a line from Ptm vertical to palatal line) and the upper border formed by sphenoid line (a line tangent to the lower border of the sphenoid bone registered on basion) [15].
(B) Oropharyngeal airway area (cm^2^): the upper border was located on the palatal line (PL); the lower border was located on the tip of the uvula line (TUL), registered on the tip of the uvula (TU), and parallel to the palatal line.
(C) Hypopharyngeal area (cm^2^): the lower border was formed by the line starting from the most anterior and superior point of the hyoid bone parallel to the palatal line (anterior of hyoid bone line, AHL) [16].

**Figure 3:**
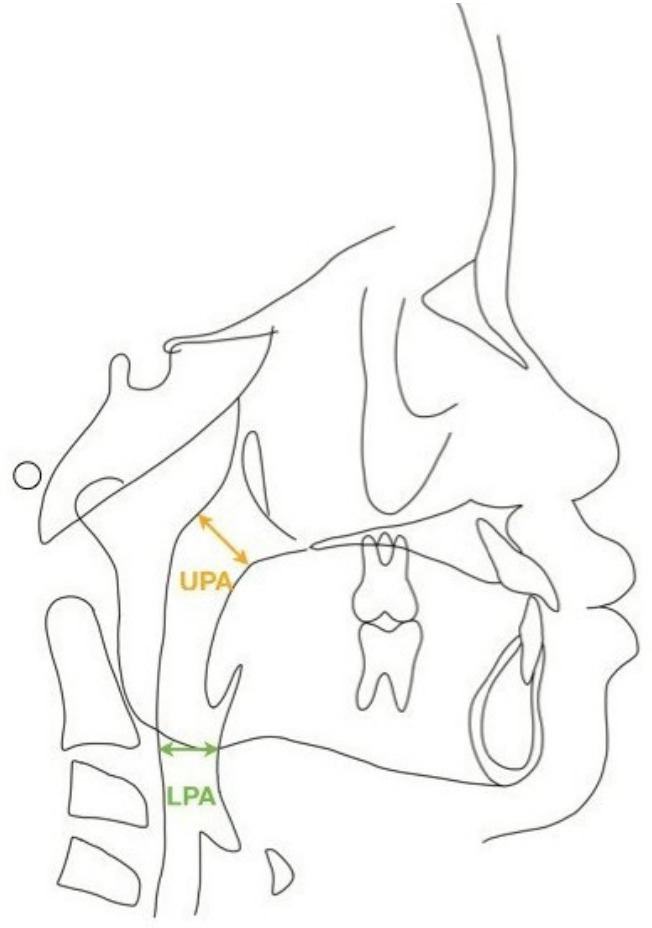
McNamara Airway Analysis for assessment of upper and lower pharyngeal airway dimensions. Upper pharyngeal airway (UPA) was measured as the distance from the upper soft palate to the nearest point on the posterior pharyngeal wall, and lower pharyngeal airway (LPA) was measured as the shortest distance from the tongue base to the nearest point on the posterior pharyngeal wall.

Linear measurements of the airway by McNamara [17] (Figure 3) were defined as:

Superior pharyngeal airway (mm): the shortest distance between the soft palate and the pharyngeal tonsil.

Inferior pharyngeal airway (mm): the shortest distance from the tongue to the nearest point of the posterior pharynx wall.

All cephalometric tracings and analyses were conducted using Dolphin Imaging Software version 7 (Patterson Dental, Chatsworth, CA, USA) by two independent examiners.

### 2.5. Reliability Assessment

To assess intraobserver reproducibility and interobserver reliability of measurements, intraclass correlation coefficients (ICCs) for the errors, along with the means and standard deviations, were calculated. A total of 20 randomly selected patient samples were taken for validation. All cephalometric landmarks were re-identified at 2-week intervals by 2 observers. The errors were analyzed by calculating the Euclidean distance between the first and second landmark coordinates. The Pearson correlation coefficient was used to calculate and validate intraobserver and interobserver reproducibility; values range from 0 to 1, with higher values indicating greater correlation or reliability.

### 2.6. Total Nasal Symptom Score (TNSS)

TNSS was used by patients to self-assess nasal symptoms. The purpose of TNSS use was to assess patients’ nasal patency before and after observation/intervention. It provides a measure of subjective complaints such as nasal obstruction, itching/sneezing, and runny nose, each rated 0–3 for a total score of up to 9 [18].

### 2.7. Statistical Analysis

A power analysis (G*Power 3.1.6; Heinrich-Heine-Universität Düsseldorf, Düsseldorf, Germany) indicated that at least 23 patients were required (α = 0.05, power = 80%). Baseline characteristics were compared using the chi-square test for categorical variables and the Mann–Whitney U test for continuous variables. Within-group comparisons were performed using the Wilcoxon signed-rank test, and between-group comparisons using the Mann–Whitney U test. Pearson correlation coefficients were used to assess intra- and inter-examiner reliability. Changes in total nasal symptom scores were analyzed using the Wilcoxon signed-rank test within groups and the Mann–Whitney U test between groups. A two-sided *p*-value < 0.05 was considered statistically significant. All analyses were performed using SPSS Statistics for Windows, Version 22.0 (IBM Corp., Armonk, NY, USA).

This study was reviewed and approved by the Institutional Review Board of Taichung Veterans General Hospital, with the approval number IRB-CG19370A.

## 3. Results

### 3.1. Baseline Data with Age, Gender, and Cephalometric Measurements in the Two Groups (Table 1)

Among the 29 children in the observation group, there were 20 boys and 9 girls, with an average age of 9.4 ± 1.4 years (112.83 ± 16.74 months, range 85–162 months), height at the 52.33 ± 25.49 percentile, and weight at the 58.30 ± 31.02 percentile. Among the 34 children in the treatment group, there were 20 boys and 14 girls, with an average age of 9.7 ± 0.68 years (116.26 ± 14.04 months, range 90–144 months), height at the 52.51 ± 28.18 percentile, and weight at the 52.33 ± 31.33 percentile. There was no statistically significant difference in gender ratio (*p* = 0.405) and average age (*p* = 0.324) between the two groups, nor was there a statistically significant difference in height percentile (*p* = 0.959) and weight percentile (*p* = 0.387). There were no statistically significant differences between the two groups regarding the nasopharyngeal, oropharyngeal, and hypopharyngeal space areas (*p* = 0.620, *p* = 0.197, *p* = 0.654), superior and inferior pharyngeal airway distances (*p* = 0.477, *p* = 0.503, respectively).

**Table 1:** Comparison of baseline data between the two groups.

Variables	Observation (*n* = 29)	Case (*n* = 34)	Effect Size	*p*-Value
Gender (*n*, %)			0.10	0.405
Male	20 (69.0%)	20 (58.8%)		
Female	9 (31.0%)	14 (41.2%)		
Age (months)	112.83 ± 16.74	116.26 ± 14.04	0.12	0.324
Height percentile	52.33 ± 25.49	52.51 ± 28.18	0.01	0.959
Weight percentile	58.30 ± 31.02	52.33 ± 31.33	0.12	0.387
Nasopharyngeal airway	3.21 ± 1.00	3.00 ± 0.84	0.06	0.620
Oropharyngeal airway	2.40 ± 0.46	2.59 ± 0.57	0.16	0.197
Hypopharyngeal airway	3.11 ± 1.13	2.98 ± 0.73	0.06	0.654
NcNamara upper	10.19 ± 3.24	9.85 ± 2.58	0.09	0.477
NcNamara lower	10.00 ± 2.69	9.75 ± 2.29	0.08	0.503

Chi-square test or Mann–Whitney U test.

### 3.2. Analysis of Differences Within Groups After One Year (Table 2)

#### 3.2.1. One-Year Airway Changes in the Observation Group

In the observation group, significant increases were observed in the oropharyngeal airway area and superior pharyngeal airway (both *p* < 0.001).

In the treatment group, significant increases were found in the nasopharyngeal, oropharyngeal, and hypopharyngeal airway areas (*p* < 0.001, *p* < 0.001, and *p* = 0.002, respectively). McNamara airway analysis also demonstrated significant increases in superior and inferior pharyngeal airway distances (*p* < 0.001 and *p* = 0.004, respectively).

#### 3.2.2. Between-Group Comparisons After One Year

Significant differences between groups were observed in the nasopharyngeal and oropharyngeal airway areas (*p* = 0.044 and *p* = 0.039, respectively). McNamara Airway Analysis showed a significant difference in upper airway distance between groups (*p* = 0.018).

#### 3.2.3. Reliability Analysis

The intraclass correlation coefficients (ICCs) for intraobserver and interobserver reliability ranged from 0.903 to 0.997 (mean 0.945) and 0.843 to 0.979 (mean 0.916), respectively, indicating excellent measurement reliability.

#### 3.2.4. Total Nasal Symptom Score (TNSS) (Table 3)

The mean TNSS change was −2.97 ± 1.30 in the treatment group and −2.00 ± 1.96 in the observation group. Significant within-group improvements were observed in both groups (*p* < 0.001 for both). However, no significant difference was found between groups (*p* = 0.078).

**Table 2:** Descriptive statistics of the airway measurements, associated *p*-values of significance tests, and effect size in the two groups.

Variables		Observation (*n* = 29)								Case (*n* = 34)							Intergroup Comparison	
	Mean Before	SD	Mean After	SD	Mean Difference	SD	Effect Size	*p*-Value ^b^	Mean Before	SD	Mean After	SD	Mean Difference	SD	Effect Size	*p*-Value ^b^	Effect Size	*p*-Value ^a^
Nasopharyngeal airway	3.21	1.00	3.51	1.15	0.30	0.80	0.19	0.294	3.00	0.84	3.54	0.95	0.54	0.62	0.67	<0.001 **	0.25	0.044 *
Oropharyngeal airway	2.40	0.46	2.81	0.81	0.41	0.66	0.58	<0.001 **	2.59	0.57	3.42	0.98	0.83	0.91	0.74	<0.001 **	0.26	0.039 *
Hypopharyngeal airway	3.11	1.13	3.56	1.09	0.45	1.14	0.36	0.050	2.98	0.73	5.14	6.91	2.17	6.91	0.54	0.002 **	0.20	0.121
NcNamara upper	10.19	3.24	11.57	3.65	1.38	2.31	0.53	<0.001 **	9.85	2.58	12.88	3.64	3.03	2.99	0.81	<0.001 **	0.30	0.018 *
NcNamara lower	10.00	2.69	10.88	3.18	0.87	3.04	0.28	0.127	9.75	2.29	11.45	3.29	1.70	3.20	0.50	0.004 **	0.15	0.235

Wilcoxon signed-rank test or Mann–Whitney U test. ^a^ Mann–Whitney U test was used for intergroup comparison. ^b^ Wilcoxon signed-rank test was used for intragroup comparison, * *p* < 0.05, ** *p* < 0.01.

**Table 3:** Total nose symptom score (TNSS) comparison between the two groups.

Variable	Observation (*n* = 29)								Case (*n* = 34)								Intergroup Comparison	
	Mean Before	SD	Mean After	SD	Mean Difference	SD	Effect Size	*p*-Value ^b^	Mean Before	SD	Mean After	SD	Mean Difference	SD	Effect Size	*p*-Value ^b^	Effect Size	*p*-Value ^a^
Rhinorrhea	2.00	0.96	1.69	0.66	−0.31	0.89	0.33	0.074	2.06	0.95	1.50	0.66	−0.56	0.66	0.65	<0.001 **	0.134	0.288
Nose stuffiness	2.47	0.76	1.66	0.72	−0.81	0.81	0.70	<0.001 **	2.47	0.72	1.38	0.60	−1.09	0.66	0.83	<0.001 **	0.160	0.204
Nose itching	1.88	1.01	1.31	0.60	−0.57	0.82	0.57	0.002 **	1.90	1.00	1.12	0.64	−0.78	0.62	0.77	<0.001 **	0.130	0.302
Nose sneezing	1.93	0.92	1.52	0.57	−0.41	1.05	0.36	0.054	1.96	0.92	1.32	0.64	−0.63	0.57	0.74	<0.001 **	0.108	0.391
Total score changes	8.17	1.89	6.17	1.26	−2.00	1.96	0.70	<0.001 **	8.26	2.03	5.29	1.53	−2.97	1.31	0.87	<0.001 **	0.222	0.078

Wilcoxon signed-rank test or Mann–Whitney U test. ^a^ Mann–Whitney U test was used for intergroup comparison. ^b^ Wilcoxon signed-rank test was used for intragroup comparison, ** *p* < 0.01.

## 4. Discussion

Among the 29 patients in the observation group and 34 in the treatment group, baseline data showed no significant differences in age, gender distribution, weight/height percentiles, or initial airway area and distance at the beginning of the intervention (Table 1).

### 4.1. Airway Changes Along with Growth

In the observation group, a one-year observation of the airway changes found that the major changes occurred in the oropharyngeal area. McNamara airway analysis revealed a significant increase in the superior pharyngeal airway distance, which is approximately correlated to the junction of the oropharynx and nasopharyngeal area. Crouse et al. evaluated the minimal cross-sectional area in normal children aged 9–11 over 1 year. Their results showed that age had a significant effect on nasal airway size, whereas gender did not. These findings were consistent with our study, which showed that the nasopharyngeal area increased with age [19]. However, the authors also noted that nasal airway size may be influenced by craniofacial growth patterns and adenoidal development, with temporary fluctuations around puberty. Chan’s cross-sectional study evaluated airway volume changes across three skeletal patterns in individuals aged 9 to 15 years. The results indicated that skeletal patterns and gender had significant effects on airway changes. Class III skeletal malocclusion group had significantly larger oropharyngeal volumes than the class I and II groups, and males had larger nasopharyngeal volumes; the total pharyngeal airway volume increased from 9 to 15 years old, with some fluctuation between ages 10 and 12 [20]. However, Chan’s study did not subclassify the total airway into nasopharyngeal, oropharyngeal, and hypopharyngeal parts. Our study indicated that patients in the observation group aged 9 to 11 years demonstrated a significant increase in the oropharyngeal and nasopharyngeal areas, but not in the hypopharyngeal area.

### 4.2. Airway Changes Due to Intervention

In the treatment group, after one year of treatment using the EF Orthoplus myofunctional appliance, significant airway changes were noted over the nasopharyngeal, oropharyngeal, and hypopharyngeal areas. McNamara Airway Analysis also revealed significant increases over the superior and inferior airway distances (Table 2). These data revealed that patients wearing the myofunctional appliance (EF orthoplus) for one year have significant airway changes. Compared with the observation-only group (Table 2), the treatment group demonstrated significant increases in the nasopharyngeal and oropharyngeal airway area and the superior pharyngeal airway distance. These results indicated that, in skeletal class II division 1 children, the effects of PMAs were not simply a push-forward effect of the mandible and tongue, as the hypopharyngeal airway did not differ between the two groups. The effects are more likely due to modification of the airway breathing pattern and to an accommodating change in the nasopharyngeal and oropharyngeal airways. However, studies comparing airway changes caused by PMAs that use observational controls are limited. Madian and Dina Elfouly’s study compared the effectiveness of airway changes of Twin Block and Myobrace appliances [16]. They found that both appliances showed improved airways. However, Twin Block was more effective than Myobrace for upper- and middle-airway improvement. Levrini et al. evaluated the effect of the Myobrace appliance in the treatment of mild-to-moderate pediatric obstructive sleep apnea (POSA). They concluded that myofunctional appliances can be an alternative to treat children with mild-to-moderate OSA. However, their observation period was short (3 months), and the sample size was small (nine patients). This preliminary study suggested that PMAs can be an alternative way of treating POSA [21].

### 4.3. Biological Mechanisms of Functional Appliances (FAs) and Prefabricated Myofunctional Appliances (PMAs) on Airway Dimensional Changes in Class II Malocclusion

Specific craniofacial characteristics that may predispose individuals to sleep-disordered breathing (SDB) include: (1) dento-craniofacial features such as anterior open bite, increased overjet, mandibular retrognathia, posterior crossbite, and a narrow, high-arched palate; and (2) facial characteristics such as a convex facial profile, extended head posture, mouth breathing, and an adenoid facies. These features are commonly observed in patients with Class II division 1 malocclusion [4].

A systematic review and meta-analysis by Xiang et al. evaluated airway dimensional changes following the use of functional appliances in growing patients with skeletal class II malocclusion [13]. Their findings demonstrated that FAs can increase upper airway dimensions, particularly in the oropharyngeal region. This effect is attributed to FAs’ ability to correct skeletal class II discrepancies through both skeletal and dentoalveolar modifications; consequently, the anatomical features that predispose individuals to pediatric sleep-disordered breathing (PSDB) may be improved.

PMAs share many structural and functional similarities with traditional custom-made functional appliances, and their mechanisms of action are considered comparable to those of FAs. However, studies evaluating airway dimensional changes in growing patients with skeletal class II malocclusion following PMA use remain limited [14,16].

### 4.4. The Use of Image Studies in Airway Measurement

The advantage of using a cephalometric radiograph as a measurement tool is its ease of use. Pediatric patients planning for PMAs are the ones who have skeletal discrepancies, such as skeletal class II or skeletal class III malocclusion. These appliances are used as phase one orthopedic correction. Therefore, comprehensive orthodontic records (study models or oral scans, extra- and intra-oral photographs, and cephalometric radiographs) and orthodontic analysis are mandatory. In our study, we used a cephalometric radiograph instead of CBCT due to age-related and radiation exposure concerns. A cephalometric radiograph is a two-dimensional image and can be used for linear and area measurements of the airway. However, the airway is three-dimensional. The correlation between 2D airway area and actual 3D airway volume warrants consideration. Aboudara et al. evaluated the correlation of 2D airway area and 3D airway volume [22]. Their results revealed a moderate-to-high correlation between airway area and volume (r = 0.75). Nguyen et al. also found a strong correlation between cephalometric films and CBCT images in assessing pharyngeal airway measurements [2]. Bronoosh’s study concluded that the pharyngeal airway area on a lateral cephalogram is correlated strongly with volumetric data on CBCT images [23]. Nowadays, even though CBCT provides more comprehensive information than two-dimensional cephalometric radiographs, cephalometric radiographs can still serve as a fundamental screening tool for providing valuable information on the morphological characteristics of the airway, as an alternative to CBCT, especially given the radiographic exposures involved for vulnerable children. In the white paper update by the American Association of Orthodontics [24], the authors mentioned the limitations of using images in the diagnosis of OSA. These included the patient’s positioning during image acquisition, nonanatomic etiology of OSA, inconsistencies in airway measurement methods, and the static nature of images versus the dynamic nature of respiration. The authors conclude that imaging using CBCT or lateral cephalograms has no diagnostic value for SDB risk assessment. In our opinion, if the measurements are combined with dynamic and functional measurements or with patient self-report outcome measures, the whole picture may become clearer.

### 4.5. Total Nasal Symptom Score (TNSS)

Total nasal symptom score (TNSS) was used in this study due to: (1) symptom severity assessment (severe nasal symptoms such as nasal obstruction would interfere with the wearing of appliance at night, in that way the compliance would be compromised); (2) treatment efficacy evaluation (in addition to image evaluations, this score provides patient self-report outcome measurements [25]); (3) reliability and validity (this score provides high internal consistency (Cronbach’s α = 0.87) and correlations with quality of life tools like RQLQ (rs = 0.77) [18]); (4) simplicity of use.

TNSS results in this study showed that all separate scores and the total score in the treatment group were significantly different, indicating that the appliance improved nasal cavity patency. However, the observation group also revealed improvements in the nasal stuffiness, itching, and total score. Even though the effect size was larger in the treatment group in all scores, the intergroup comparison did not reveal significant differences.

### 4.6. Strengths, Limitations, and Future Perspectives

The strengths of this study were: (1) Although this was a retrospective evaluation, observation-only controls with the same kind of malocclusion, age range, gender ratio, growth percentile, and weight percentile were used as controls. (2) Patient self-report measurement was used (TNSS). This provided the clinician with a clear view of the patient’s subjective perception of the changes in the nasal patency, in addition to the objective data record.

The limitations of this study were: (1) The retrospective nature of this study. (2) Using two-dimensional images for airway assessment instead of three-dimensional CBCT or MRI images. (3) TNSS represents nasal patency only. The sleep breathing problems may involve anatomic features such as adenoid, tonsil, and other nonanatomic factors. (4) Limited sample size and observation period.

While preliminary clinical and radiographic studies in our study indicate potential benefits, the long-term stability and clinical significance of these airway modifications remain areas of ongoing research. Future studies will adopt more dynamic and functional assessments, enhancing our understanding of the dynamic relationship between airways and breathing.

## 5. Conclusions

Prefabricated myofunctional appliance therapy is associated with significant improvements in airway dimensions, particularly in the nasopharyngeal and oropharyngeal regions, in children with class II division 1 malocclusion. These findings suggest that PMAs may promote functional airway adaptation beyond simple mandibular advancement. Further prospective studies with comprehensive functional assessments are needed to confirm the clinical significance and long-term stability of these changes.

## Data Availability

The data that support the findings of this study are available from the corresponding author upon reasonable request.
